# The Journey to smORFland

**DOI:** 10.1002/cfg.325

**Published:** 2003-10

**Authors:** Wagied Davids, Hans-Henrik Fuxelius, Siv G. E. Andersson

**Affiliations:** Department of Molecular Evolution Evolutionary Biology Center Uppsala University Norbyvägen 18C Uppsala 752 36 Sweden

## Abstract

The genome sequences completed so far contain more than 20 000 genes with unknown function and no similarity to genes in other genomes. The origin and evolution of the orphan genes is an enigma. Here, we discuss the suggestion that some orphan genes may represent pseudogenes or short fragments of genes that were functional in the genome of a common ancestor. These may be the remains of unsuccessful duplication or horizontal gene transfer events, in which the acquired sequences have entered the fragmentation process and thereby lost their similarity to genes in other species. This scenario is supported by a recent case study of orphan genes in several closely related species of *Rickettsia*, where full-length ancestral genes were reconstructed from sets of short, overlapping orphan genes. One of these was found to display similarity to genes encoding proteins with ankyrin-repeat domains.


**Comparative and Functional Genomics**
*Comp Funct Genom* 2003; **4**: 537–541.
Published online in Wiley InterScience (www.interscience.wiley.com). **DOI:** 10.1002/cfg.325

**Conference Review**

### The journey to smORFland

Wagied Davids, Hans-Henrik Fuxelius and Siv G. E. Andersson*
*Department of Molecular Evolution, Uppsala University, 752 36 Uppsala, Sweden*

**Correspondence to:
Siv G. E. Andersson, Department of Molecular Evolution, Evolutionary Biology Center, Uppsala University, Norbyvägen 18C, 752 36 Uppsala, Sweden.
E-mail: siv.andersson@ebc.uu.se;
URL: http://artedi.ebc.uu.se/molev*

Received: 29 May 2003
Revised: 6 August 2003
Accepted: 6 August 2003

### Abstract

**The genome sequences completed so far contain more than 20 000 genes with unknown function and no similarity to genes in other genomes. The origin and evolution of the orphan genes is an enigma. Here, we discuss the suggestion that some orphan genes may represent pseudogenes or short fragments of genes that were functional in the genome of a common ancestor. These may be the remains of unsuccessful duplication or horizontal gene transfer events, in which the acquired sequences have entered the fragmentation process and thereby lost their similarity to genes in other species. This scenario is supported by a recent case study of orphan genes in several closely related species of *Rickettsia*, where full-length ancestral genes were reconstructed from sets of short, overlapping orphan genes. One of these was found to display similarity to genes encoding proteins with ankyrin-repeat domains. Copyright © 2003 John Wiley & Sons, Ltd.**

### Introduction

It is postulated that one-third of every sequenced genome contains open reading frames (ORFs) with no known function [1,2]. These are either classified as 'hypothetical' if no sequence homologues are found in the public databases, or 'hypothetical conserved' if they display similarity to genes that also have no identified function. The hypothetical ORFs without sequence homologues appear in the literature under a variety of names; most commonly, the term 'orphan' is used, but they are also referred to as ORFans [2,3] or ELFs (evil little fellows) [4]. The uncertainty about whether they correspond to real genes complicates estimates of total gene numbers. A related challenge is to identify very small, functional genes (<100 codons), which are difficult to distinguish from small, meaningless ORFs [5]. In this paper, we visit the so-called smORFland (**http://smorfland.microb.uni.wroc.pl/**) that is inhabited by lone or small ORFs without a functional partner.

Ever since their discovery, orphan genes have been a mystery to genome annotators with their enigmatic presence and mode of evolution remaining as yet unresolved. Initially, it was thought that the number of genes with unknown function would rapidly decrease with the increasing number of completed genomes; however, this does not seem to be the case. Instead, traditional methods employed during genome annotation consistently fail to find evolutionary relatives for orphan genes based solely on sequence similarity to known homologues in the current databases. This raises questions such as: if sequence conservation is meant to imply evolutionary relatedness, why then do these orphan sequences fail to find their relatives?; or simply, do none exist?

### The slippery slope of orphan gene identification

The annotation of ORFs that are not classified by similarity searches against public databases represents an exponentially growing problem. Not only do their functions remain to be determined by experimental methods, it is also often difficult to verify that they correspond to protein-coding genes. One of the challenges is that orphan genes appear to be shorter on average than genes for which orthologues are identified in other systems [6,7]. For

Copyright © 2003 John Wiley & Sons, Ltd.

example, orthologous genes in *Rickettsia conorii* and *Rickettsia prowazekii* have an average length of 1030 bp, whereas orphan genes that are solely present in *R. conorii* exhibit a much shorter average length of only 313 bp [8]. The difficulty with these and other short sequences is to obtain reliable estimates of various gene statistics, such as codon usage biases that are normally useful for discriminating coding from non-coding sequences.

At the beginning of the genome era when only a few microbial genomes had been fully sequenced and annotated, it was clear that about 20–30% of the identified ORFs could not be matched against any known sequences in the standard databases [1]. As more genome sequence data became available, some of the orphans in the earlier sequenced genomes found matches in these new genomes and hence are no longer referred to as orphans. A fraction of orphans is specific for strains, another for species and yet another is unique at the level of the genus. For each newly sequenced genome, more orphans are added to the 'orphan-space' than are being resolved, which means that the total number of orphans is steadily growing. For example, the addition of sequence data from closely related genomes will lower the previous set of species-specific orphans; however, the number of genus-specific orphans will stay the same or even increase, due to the addition of a new set of species-specific orphans.

Currently, it is estimated that the number of families containing one or more orphan genes is well over 20 000 [2]. This is based on an analysis of 60 sequenced genomes in which genes were added one by one and tagged as either orphans or non-orphans. As soon as an ORF in one genome matched an ORF in one of the already analysed genomes, the latter was re-tagged as a non-orphan. The same procedure was iterated through all of the 60 genomes. In total, the number of ORFs was 168 248 and the number of ORFans was 23 634, which is 23% of the data set [2]. If it is assumed that all represent real genes, it can be estimated that more than 25 000 orphan genes will await functional and structural classification after the 100th genome. But are these ORFs really genes?

### From where do the orphan genes originate?

At first glance, orphan genes were thought to be artefacts that would vanish as more genome sequences became available. However, as discussed above, orphans are here to stay. So, why is it that so many genes show no similarity to other genes in the databases, and where do they all come from? If all genomes and their genes were descendants of one common unifying ancestor, there has to be a reasonable explanation for the high numbers of genes that seem to have arisen from nowhere. Below, we outline a few possible scenarios for how orphans might have originated.

First, it is almost certainly the case that a certain fraction of orphan genes simply represents incorrect gene annotations [6]. In genomes with unbiased nucleotide frequencies, it is very difficult to distinguish ORFs that correspond to real genes from ORFs that occur by chance, particularly in cases of very short ORFs. In addition, some genes may code for structural proteins with no, or weak, selection for amino acid content or composition; these are expected to evolve very fast at the amino acid level and may quickly become unrecognizable by standard database search methods [9].

In addition, it seems reasonable to assume that at least some orphans represent pseudogenes or short gene fragments. In particular, rapidly evolving segments of genes undergoing fragmentation may easily be misdiagnosed as orphans. Vertically transmitted single gene copies may end up as orphans if selection for the original gene function was lost, e.g. due to altered environmental conditions (Figure 1). Orphans may also be derived from gene fragments of highly divergent members of large protein families (Figure 1), or they may have arisen from the inflow of external DNA via horizontal gene transfer (Figure 2).

In the latter two cases, the selective constraints acting on the acquired genes will often be of only transient nature. Following loss of selection, the duplicated and horizontally acquired genes will become inactivated and start deteriorating, a process during which short segments of the ancestral genes may temporarily remain. For such short gene segments, only those that correspond to highly conserved functional domains will be recognizable by sequence similarity searches. In contrast, segments that correspond to non-conserved parts will not necessarily find their sequence homologues in the public databases and these may easily be misclassified as orphans (Figure 1). Occasionally, however, merging fragments of deteriorating genes may result in an orphan sequence with a novel gene

Copyright © 2003 John Wiley & Sons, Ltd.

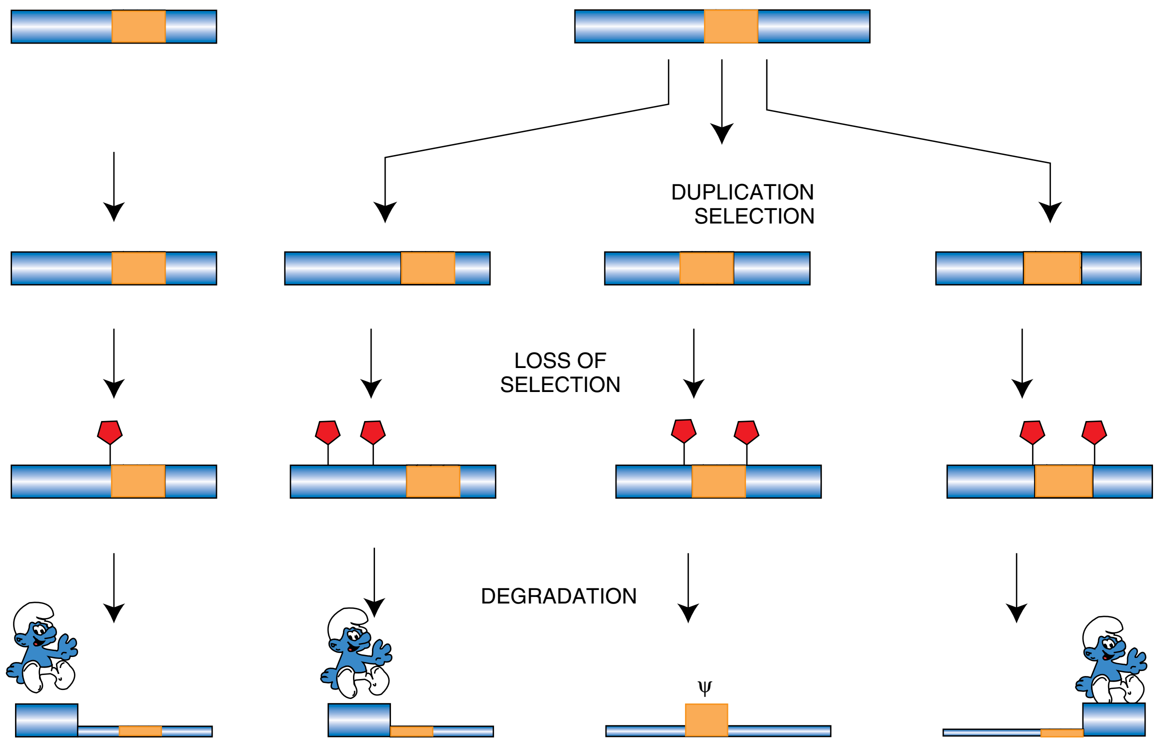

**Figure 1.** Orphans may be derived from gene fragments of single-copy genes as well as from highly divergent members of large protein families. Following loss of selection and degradation, ORFs in non-conserved segments of the gene fail to find their sequence homologues in other species. Yellow and blue boxes correspond to conserved vs. non-conserved gene fragments, respectively. Red hexamers show the position of stop codons and $\psi$ refers to pseudogene fragments for which sequence similarity to other genes can be recognized. The smorfs highlight the locations of orphans originating during the fragmentation process function. The difficulty is how to distinguish one from the other.

### How to find out?

Gene-specific sequence patterns and nucleotide frequency statistics are normally defined from analyses of known genes, and these measures are then used to search for unknown, novel genes. In the case of orphans that result from incorrect annotations of randomly occurring ORFs, the development of more sophisticated statistical methods for codon usage analysis may facilitate the annotation process. However, for orphans that are derived from pseudogenes and gene fragments, this does not necessarily represent a way forward, since the gene fragments may have retained most of the nucleotide patterns of their full-length, ancestral gene. Thus, in terms of sequence patterns this category of orphans may look just like real genes, at least during an early phase of the degradation process.

Another method for assessing the protein-coding potential of genomic regions is by estimating the degree of sequence conservation, provided that two or more closely related sequences are available for comparison. This approach takes advantage of the fact that in the vast majority of coding regions, non-synonymous substitutions ($K_A$) that affect amino acid contents occur at much lower frequencies than synonymous substitutions ($K_S$), which do not effect protein compositions [12]. If selection on protein function is released, such as in pseudogenes and degrading gene fragments, non-synonymous sites will start accumulating mutations at the same high frequencies as synonymous sites.

This approach was used by [4] to distinguish short orphans from authentic protein-coding genes. The same approach was also taken to show that gene fragments in *Rickettsia conorii* accumulate

Copyright © 2003 John Wiley & Sons, Ltd. 

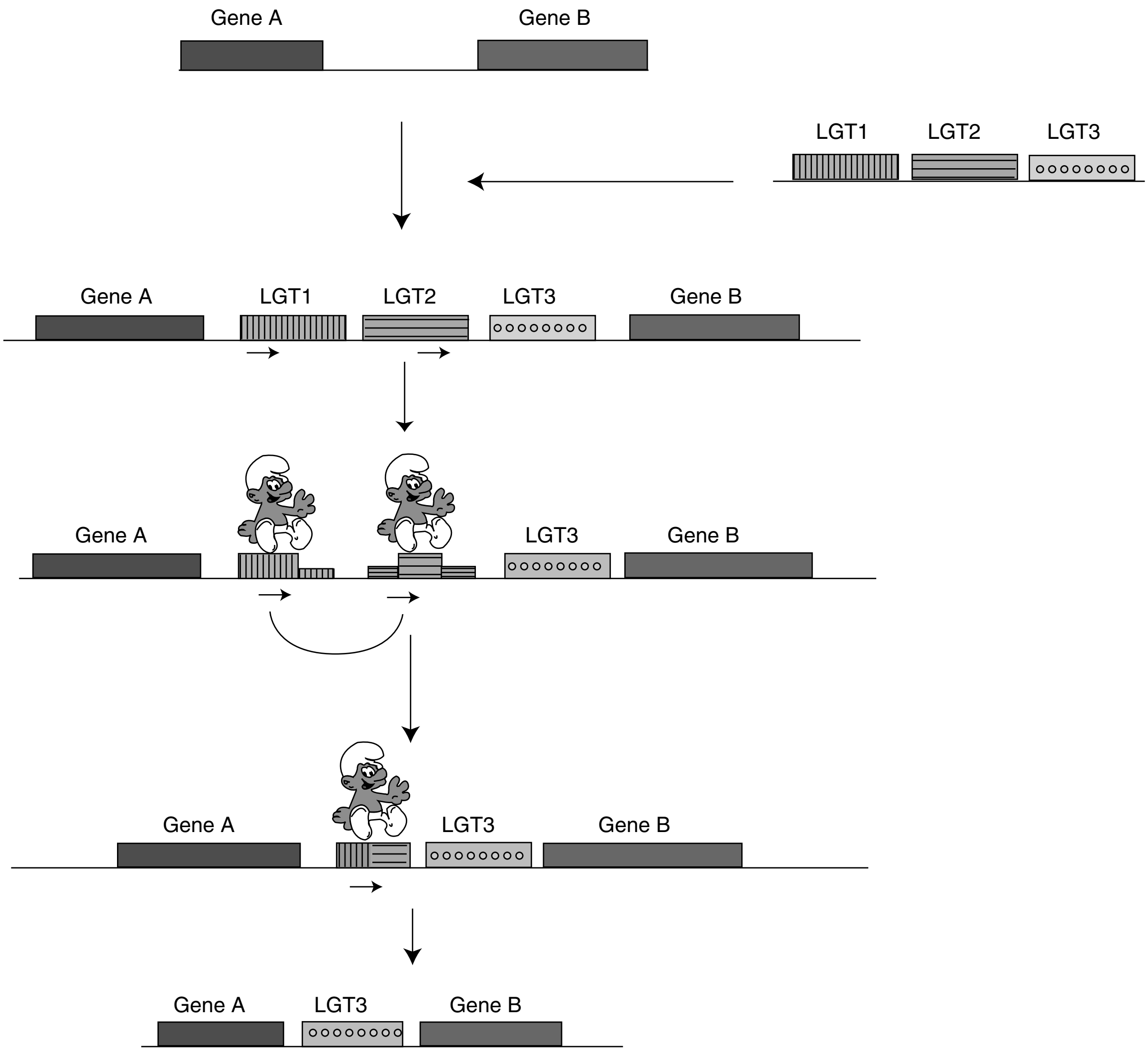

**Figure 2.** Orphans may be derived from fragmented genes that were acquired via horizontal gene transfer. Selected genes (LGT3) will be retained, whereas segments that offer no selective advantage (LGT1 and LGT2) will be rapidly lost, sometimes via recombination at repeated sites (arrows). Gene A and gene B represent conserved, functional genes. The smorfs highlight the locations of orphans originating during the fragmentation process mutations at similar frequencies, irrespective of whether or not they produce RNA [10]. However, one note of caution is that ORFs with high $K_A$:$K_S$ ratios may not necessarily be non-coding, since genes encoding structural proteins and/or pili may also accumulate mutations at high frequencies [9].

### Once upon a time there was an ankyrin-repeat protein. . .

We have used *Rickettsia* as a model system for studies of the origin and fate of orphan gene sequences. As many as 412 orphans were identified in the 1.3 Mb genome of *Rickettsia conorii* [11] and these are randomly distributed around the genome. If they represent remnants of full-length genes that once had identifiable functions, it is of interest to reconstruct these genes and try to infer their ancestral functions.

To this end, we have reconstructed the putative full-length ancestral protein-coding sequences from a set of short, overlapping orphan genes in multiple, closely related *Rickettsia* genomes and searched for evidence of their ancestral functional

Copyright © 2003 John Wiley & Sons, Ltd. 

status [8]. The analysis was based on a phylogenetic approach that considered both comparative protein sequence and structure information [8]. Interestingly, comparative homology protein modelling revealed that one of the reconstructed full-length ancestral genes displayed structural similarity to the consensus ankyrin repeat domain [8]. Indeed, it seems likely that the common ancestor of *Rickettsia* should have encoded such proteins, since more than 20 highly divergent ankyrin-repeat containing genes have been identified in the genome of its close relative *Wolbachia pipientis*. This provides strong evidence for the hypothesis that short orphans represent remnants of genes that were once functional.

### Conclusions and future perspectives

Recently, orphans have come into the spotlight because they are putative targets in the search for novel gene functions and therefore of particular interest for massive functional and structural analyses. So far, the use of comparative sequence approaches in the study of orphans has revealed a fascinating interplay between coding and non-coding sequences over time. However, the disappointing message from these exercises is that not all orphans correspond to real genes. Some were incorrectly identified as genes and others correspond to degraded gene fragments. The challenge for the future will be to distinguish all the various kinds of orphans and carefully select those that are most likely to correspond to real genes for further experimental studies.

Here, a case study of *Rickettsia* offers promising insights. In this species, a large majority of orphans represent short gene fragments. The analysis showed that full-length ancestral gene sequences could be reconstructed from extant gene remnants of very closely related species [8]. This represents a novel approach to validate the authenticity of the ancestral genes, and to gain insights into the mechanisms and modes of sequence evolution of these gene vagrants. The ubiquitous and widespread occurrence of orphans indicates that a high rate of sequence turnover is part of the normal mutational engine that generates much of the genomic variability that we observe. The extreme cases of conservation observed for some genes, such as the rRNA genes, represent exceptions that, if handled correctly, are useful for tracing species histories.

To support reconstructions of ancestral gene sequences in a large-scale manner from multiple, closely related genomes, fully automated methods for ancestral gene reconstruction need to be developed. Once these tools are in place, we will be able to analyse the functions and structures of rare and extinct genes in the same broad, systematic manner as is currently done for contemporary genes.

### References

1. Fraser CM, Eisen JA, Salzberg SL. 2000. Microbial genome sequencing. *Nature* **406**: 799–803.
2. Siew N, Fischer D. 2003. Twenty thousand ORFan microbial protein families for the biologist? *Structure* **11**: 7–9.
3. Fischer D, Eisenberg D. 1999. Finding families for genomic ORFans. *Bioinformatics* **15**: 759–762.
4. Ochman H. 2002. Distinguishing the ORFs from the ELFs: short bacterial genes and the annotation of genomes. *Trends Genet* **18**: 335–337.
5. Basrai M, Hieter P, Boeke J. 1997. Small open reading frames: beautiful needles in the haystack. *Genome Res* **7**: 768–771.
6. Skovgaard M, Jensen JL, Brunak S, Ussery D, Krogh A. 2001. On the total number of genes and their length distribution in complete microbial genomes. *Trends Genet* **17**: 425–428.
7. Mira A, Klasson L, Andersson SGE. 2002. Microbial genome evolution: sources of variability. *Curr Opin Microbiol* **5**: 506–512.
8. Amiri H, Davids W, Andersson SGE. 2003. Birth and death of orphan genes in *Rickettsia*. *Mol Biol Evol* (in press).
9. Lawrence J. 2003. When ELFs are ORFs, but don't act like them. *Trends Genet* **19**: 131–132.
10. Davids W, Amiri H, Andersson SGE. 2002. Small RNAs in Rickettsia: are they functional? *Trends Genet* **18**: 331–334.
11. Ogata H, Audic S, Renesto-Audiffre P, *et al*. 2001. Mechanisms of evolution in *Rickettsia conorii* and *R. prowazekii*. *Science* **293**: 2093–2098.
12. Nekrutenko A, Makova K, Li W. 2001. The $K_A/K_S$ ratio test for assessing the protein-coding potential of genomic regions: an empirical and simulation study. *Genome Res* **12**: 198–202.

Copyright © 2003 John Wiley & Sons, Ltd.
